# Mental Health and Well-Being of Residents with Parkinson’s Disease in Care Homes: A Scoping Review

**DOI:** 10.3390/healthcare13212791

**Published:** 2025-11-04

**Authors:** Arnelle Gillis, Gary Mitchell, Stephanie Craig

**Affiliations:** School of Nursing and Midwifery, Queen’s University Belfast, Belfast BT9 7BL, UK; gary.mitchell@qub.ac.uk

**Keywords:** Parkinson’s Disease, mental health, care homes, depression, anxiety, well-being

## Abstract

Introduction: As the prevalence of Parkinson’s disease (PD) increases, care homes face growing challenges in managing residents’ complex mental health needs. Residents may experience low mood, anxiety, and hallucinations. Methods: A scoping review (ScR) was conducted following the methodological framework of Arksey and O’Malley and reported according to the PRISMA-ScR checklist. Four databases (CINAHL, Embase, PsycINFO, and Scopus) and grey literature sources were searched up to June 2025, alongside grey literature (European Public Health Association (EUPHA), the UK Department of Health and Social Care, the National Institute for Health and Care Excellence (NICE), and the World Health Organisation (WHO), with the aim of mapping the existing evidence on the mental health and well-being of people living with Parkinson’s disease in care homes, in order to identify gaps in the literature. Screening and data extraction were conducted independently by two reviewers using Covidence software, with discrepancies resolved through discussion. Results: Eleven studies met the inclusion criteria, encompassing quantitative, qualitative, and mixed-methods designs. The findings indicate that mental health disorders are common and severe among care home residents with PD; 61% of individuals experienced at least one, contributing to reduced quality of life, increased care dependency, emotional distress, and social isolation. Caregivers report significant burden associated with managing symptoms associated with mental health disorders and residents frequently experience a decline in psychosocial well-being. Conclusions: Although few studies evaluated interventions, the findings highlight the need for both pharmacological and non-pharmacological approaches. Specialised staff training and adherence to international care guidelines are needed to improve recognition and management of mental health needs in residents living with PD.

## 1. Introduction

Parkinson’s disease (PD) is a progressive neurodegenerative disease primarily characterised by motor symptoms such as tremor, rigidity, and bradykinesia [1]. More than 10 million people worldwide are affected, making PD a significant challenge for healthcare systems globally [2]. In advanced stages, many individuals require round-the-clock support and ultimately die in institutional settings such as care homes or hospitals [3]. PD predominately affects older adults with most individuals (71%) aged 65 years or over, and gender distribution shows that it is more common in men [4].

As the disease progresses, non-motor symptoms (NMS) become increasingly burdensome, with neuropsychiatric symptoms (NPS) having a profound impact on quality of life [1]. NPS include anxiety, hallucinations, and apathy, which are often grouped under the broader category of mental health symptoms in clinical and research literature. In this review, the term mental health symptoms are used as an umbrella concept to capture the full range of mental health experiences people with PD in care homes have. While NPS is the more common term in PD-specific research, the term mental health symptoms is preferred here for its wider relevance to caregiving, nursing practice, and policy. Both terms are used where appropriate, with NPS understood as a subset of the broader mental health symptomatology under consideration.

Recent research shows that the economic burden and medical resource use in PD increases as the disease progresses, with a three-fold increase in annual healthcare utilisation in advanced cases and therefore a greater reliance on hospitalisations, prescription medications, and complex caregiving tasks, subsequently placing substantial pressure on both caregivers and institutions such as care homes [4]. The term “care home” refers to facilities that provide 24 h care and supervision, typically for older adults with complex care needs. These include both nursing homes, where care is provided or overseen by registered nurses, and residential care homes, which focus on personal and social care without on-site nursing [5]. Terminology varies internationally: in Australia, these are often called Residential Aged Care Facilities (RACFs), and in the United States, the closest equivalent is nursing home or long-term care facility. Importantly, care homes differ from assisted living facilities, home care services, or rehabilitative skilled nursing facilities, as those typically offer short-term or lower-intensity support [6]. In this review, the term care home encompasses settings that provide permanent, full-time care, whether nursing-led or residential for individuals who can no longer be safely supported in their own homes.

The psychological stress of living with a chronic disease like PD can mean residents are at a higher chance of developing depression [7]. Depression in PD also reflects neurobiological changes, including disruption of dopaminergic and serotonergic pathways and limbic–cortical circuits; so both disease biology and psychosocial stressors contribute.

Research by Lacy et al., 2023 [8] highlighted that depressive symptoms like low mood can impair quality of life more severely than even advanced motor symptoms. Mental health, although less visible to carers and nursing staff, can significantly affect a patient’s daily functioning. Caregiving responsibilities often shift to family members or informal caregivers when cognitive decline and worsening motor function demand more intensive care [9]. Caregivers report that managing anxiety, depression, and cognitive decline in residents with Parkinson’s disease is one of the most challenging aspects of care, which in turn can affect the quality of care provided [10]. During the COVID-19 pandemic, with restrictions on healthcare access, the importance of adaptable, responsive care strategies to maintain quality of life during periods of crisis was highlighted [11]. The admission of a person with PD into a care home adds a layer of complexity, as staff often prioritise physical care needs, with mental health and well-being frequently overlooked [12]. Unfortunately, social isolation can be a domino effect for residents experiencing anxiety; therefore, it is important to establish supportive relationships that can help to promote a positive quality of life [13]. A systematic review reported that depression is a prevalent disorder in individuals with PD, affecting between 44% and 51.7% of residents [14]. Depression and symptoms related to this common disorder of low mood are often under-recognised by clinicians, as they can overlap with other features of PD, such as reduced facial expression, decreased appetite, and insomnia [15]. This underreporting highlights the importance of integrated care plans that address both physical and psychological needs [16].

## 2. Materials and Methods

### 2.1. Aim

Aim: To systematically map the extent, range, and nature of research on mental health and well-being among residents with Parkinson’s disease in care home settings.

### 2.2. Study Design

A scoping review (ScR) was conducted following the methodological framework proposed by Arksey and O’Malley [17] and guided by the Preferred Reporting Items for Systematic Reviews and Meta-analyses extension for Scoping Reviews (PRISMA-ScR) checklist [18]. This process included the development of a clear research question, the formulation of eligibility criteria, and the systematic identification and selection of relevant studies. Data were extracted from the included studies and synthesised to map the breadth and nature of the available evidence. A scoping review protocol was registered at (https://osf.io/ebrw6/, accessed on 1 November 2025). 

### 2.3. Study Selection

Scoping reviews often deal with broad complex topics. The population–exposure–outcome (PEO) framework helps narrow the focus whilst allowing for flexibility [19]. A comprehensive literature search was conducted in June 2025, employing a combination of medical subject headings (MeSH) and relevant free text, including “Parkinson Disease,” “care homes,” and “Depression,” as detailed in Table 1. Variants of the term “Parkinson’s disease” were included to capture the full spectrum of relevant terminology used across the academic literature. Keywords related to care homes and mental health were selected because of the diverse range of mental health-associated terminology. Variations pertaining to care homes were included to identify studies focusing on nursing homes and residential care settings. Truncation wildcards (*) were applied to key search terms to retrieve alternative word forms and enhance search sensitivity.

Four electronic databases, CINAHL, Embase, PsycINFO, and Scopus, were searched to June 2025. These databases were purposefully selected for their relevance and coverage across the research topics. CINAHL was included for its strong emphasis on nursing and allied health literature, making it particularly valuable for identifying studies related to clinical care. Embase offers a broad range of biomedical and pharmacological research, thereby ensuring the inclusion of relevant scientific studies. PsycINFO, with its specialisation in psychology and mental health, captures literature related to psychiatric and neuropsychiatric dimensions. Scopus, as a multidisciplinary database, contributed to the breadth and diversity of the literature. In addition to peer-reviewed sources, grey literature was also searched through targeted searches of the European Public Health Association (EUPHA), the UK Department of Health and Social Care, the National Institute for Health and Care Excellence (NICE), and the World Health Organisation (WHO). Reference lists of all included studies were manually reviewed to locate additional studies not retrieved through database searches. Internet-based searches were also conducted using Google and Google Scholar to identify further peer-reviewed literature. Example CINAHL Boolean line: (“Parkinson disease” OR Parkinson OR parkinsonism) AND (“nursing home” OR “care home*” OR “residential home*” OR “long-term care”) AND (“depression” OR “anxiety” OR “psychosis” OR “hallucination*” OR “well-being”). 

### 2.4. Criteria for Inclusion

The academic literature focuses on the mental health and well-being of individuals living with PD in care home settings. For this review, ‘care homes’ refer to institutional, 24 h residential or nursing facilities providing permanent care, whereas ‘community-based settings’ refer to home care, day centres, or outpatient services where individuals continue living independently. This distinction avoids overlap between residential and community contexts. To ensure a broad and inclusive evidence base, studies were eligible if they reported data from caregivers of individuals with PD. This approach allowed for a wider range of perspectives on the effects of mental health. In studies that included participants with PD alongside other conditions, only data specific to PD were extracted. No restrictions were applied regarding publication date. The inclusion and exclusion criteria are summarised in Table 2. Only English-language studies were included due to limited translation resources. While this restriction may have excluded some relevant international evidence, it ensured feasibility and consistency in data extraction and quality appraisal.

### 2.5. Data Extraction

All records were imported into Covidence software [20], resulting in 1192 identified records. Covidence (https://www.covidence.org/) is a web-based software platform designed to streamline systematic and scoping review workflows, including citation import, automatic duplicate removal, title/abstract screening, full-text review, and data extraction. It allows multiple reviewers to work independently and tracks all inclusion/exclusion decisions for transparency. Covidence automatically removed 339 duplicates, leaving 853 unique records for screening. Title and abstract screening were conducted independently by two reviewers (AG and GM), with both screening 100% of records to ensure consistency and reduce bias. Discrepancies were resolved through consultation with a third reviewer (SC). GM is a Reader in Nursing with experience in evidence synthesis, care homes, and dementia research, and has authored extensively in these fields. SC is Lecturer in Nursing, specialising in care of older people, with expertise in scoping and systematic reviews. AG is a geriatric nurse specialist and has contributed to three peer-reviewed publications. Collectively, the team brings complementary expertise in nursing, care homes, and evidence synthesis.

**Figure 1:**
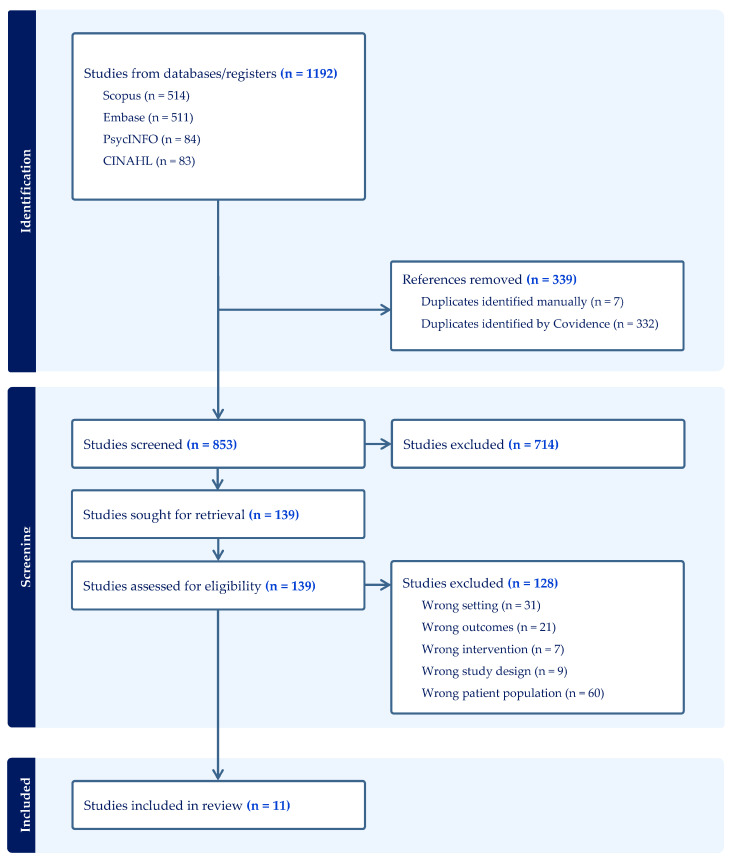
PRISMA flow diagram.

Following this, 139 records were deemed potentially eligible and retrieved for full-text review, which was also conducted independently by AG and GM. As before, any disagreements were resolved through discussion with SC to ensure accuracy and consensus. After applying the predefined inclusion and exclusion criteria, 128 studies were excluded, leaving 11 remaining included studies. The most common reasons for exclusion were wrong setting (n = 31), wrong outcomes (n = 21), wrong intervention (n = 7), wrong study design (n = 9), and wrong patient population (n = 60). The full study selection process is illustrated in Figure 1 (PRISMA flow diagram).

### 2.6. Data Analysis

Thematic analysis was used in this scoping review to identify and interpret recurring ideas and concepts within the data, guided by Braun and Clarke’s six-phase framework [21]. AG led the analysis, repeatedly reviewing the extracted data to develop a detailed understanding of its content and context. Initial reflections and emerging patterns were documented during this familiarisation phase. AG and GM then looked through the data and gave labels to parts that were important for answering the review’s aim. These codes were reviewed collectively to identify relationships and form preliminary groupings, which served as potential themes. Themes were refined through a process of collapsing overlaps, checking consistency, and ensuring alignment with the research questions.

Once finalised, themes were clearly defined and supported by illustrative data extracts, forming the basis of a narrative synthesis that captured the scope and nature of each theme. AG conducted the analysis with regular input from the broader review team, who provided ongoing feedback and quality assurance. The quality of the included studies was also considered, drawing on the Mixed Methods Appraisal Tool Version 2018 (MMAT) [22], alongside sample size, methodological rigour, and consistency of findings across studies.

## 3. Results

### 3.1. Characteristics of Included Studies

A total of 11 studies met the inclusion criteria which includes studies published between 1999 and 2025 [23,24,25,26,27,28,29,30,31,32,33]. These studies employed a variety of research designs, including quantitative, qualitative, and mixed-methods approaches. The participants in these studies were individuals diagnosed with PD, many of whom were in the later stages of the disease, along with caregivers, family members, and healthcare professionals. These studies were conducted in a range of settings, including nursing homes and care homes, across several countries.

Among the 11 studies, 7 used quantitative research designs [23,24,25,31,32,33,34], such as non-randomised observational or descriptive studies. These studies were conducted in various institutional settings, involving large sample sizes ranging from 73 to 692 participants. The quantitative studies typically employed structured assessments and standardised scales to gather data. For example, Weerkamp et al., 2013 [23] conducted a cross-sectional study in the Netherlands with 73 nursing home residents to assess the prevalence of mental health symptoms in individuals with PD. Other studies, such as those by Aarsland et al., 1999 [24] and Hosking et al., 2020 [25], employed structured interviews and surveys to gather data on mental health symptoms, clinical characteristics, and treatment complications, with sample sizes of 139 and 692 participants, respectively. In contrast, three studies employed qualitative research designs [26,27,29], focusing on structured interviews and focus group discussions to explore the lived experiences and perceptions of people with PD, their caregivers, and healthcare providers. These studies had sample sizes generally smaller than those of the quantitative studies. For instance, the study by Kurtgöz et al., 2024 [26] in Turkey involved just eight participants, while van Rumund et al., 2014 [27] conducted semi-structured interviews with 15 residents and 15 informal caregivers, alongside focus groups with healthcare professionals. These qualitative studies aimed to gain a deeper understanding of the emotional, spiritual, and practical challenges faced by individuals with PD and those who care for them. One study, by Lex et al., 2018 [28], employed a mixed-methods approach, integrating both qualitative and quantitative data collection methods.

The sample sizes of the studies varied considerably. The largest sample size was 692 participants in the study by Hosking et al., 2020 [25], which compared late-stage PD patients in care homes with those living at home. On the other hand, the smallest sample size was eight participants in the qualitative study by Kurtgöz et al., 2024 [26], which explored the spiritual care needs of elderly PD patients and caregivers in care homes. Ethical considerations were consistently addressed across all studies, with each study obtaining ethical approval. Most studies also described clear processes for informed consent, ensuring that participants were fully aware of the study’s objectives and their rights. Informed consent procedures were carefully followed, with researchers ensuring participant confidentiality and data protection throughout the research process. Table 3 provides a summary of the review findings.

### 3.2. Quality Appraisal

The methodological quality of the studies included in this review was assessed using the MMAT [22], a validated framework for evaluating qualitative, quantitative, and mixed-methods research designs. All studies met the MMAT’s initial screening criteria and were subsequently appraised across five domains, including clarity of research objectives, appropriateness of recruitment strategies, suitability of data collection methods, and analytical rigour. Of the included studies, six received the highest score of 5/5, indicating strong methodological quality with no notable limitations [25,27,29,30,31,32]. The remaining five studies were rated 4/5, reflecting generally robust methods but with minor limitations, such as incomplete reporting of sampling procedures or limited integration of mixed-methods components [23,24,25,27,33].

### 3.3. Results: Synthesis of Evidence

There were three key themes regarding the mental health and well-being of residents with PD in care home settings. Three overarching themes were identified across the included studies. Theme 1 outlines the range and nature of mental health problems experienced by residents with Parkinson’s disease in care homes. Theme 2 explores why these problems often persist, highlighting residents’ experiences of unmet emotional and psychological needs. Theme 3 examines the organisational and systemic factors that shape how mental health support is delivered within care home settings. Together, these themes provide an integrated overview of the evidence on mental health and well-being among this population.

#### 3.3.1. Theme 1: Complex Mental Health

Mental health disorders, particularly depression, anxiety, and psychosis, are highly prevalent among people living with Parkinson’s disease. Depressive symptoms, such as low mood, are understood as components of depressive disorder rather than as separate conditions. These non-motor manifestations often occur alongside the recognised motor symptoms of Parkinson’s disease, including tremor, rigidity, and bradykinesia. This theme synthesises evidence from the included studies to show how complex mental health problems influence the emotional and social well-being of residents with Parkinson’s disease in care homes.

Early studies laid the groundwork for understanding the mental health burden in PD, with Aarsland et al. 1999 [24] among the first to document the range of symptoms in people living with PD in care homes using the neuropsychiatric inventory (NPI). This study reported that 61% of individuals with Parkinson’s disease exhibited at least one symptom or disorder; depression (38%) and hallucinations (27%) being the most common. These findings were seminal in shifting the clinical focus from purely motor symptoms to recognise that poor mental health impacts residents’ daily lives.

Subsequent studies have corroborated these early findings. For instance, Chang et al. 2023 [30], in a nationwide observational study which took place in Taiwan, reported that the prevalence of depression in the study was similar to previous cross-sectional studies at 14.3%, with high rates of anxiety and hallucinations also observed. It was also contributing to their declining functional abilities. Similarly, Weerkamp et al., 2013 [23] investigated mental health problems in nursing home residents with Parkinson’s disease and found that the overall non-motor symptom burden was strongly associated with poorer quality of life, explaining 45% of its variance (R^2^ = 0.45). This finding highlights that mental health symptoms can be as disabling as motor impairments.

Cognitive decline in PD is often accompanied by mood disorders and psychiatric disturbances, which together further impair communication, decision-making, and memory. Pigott et al., 2023 [31] emphasised that residents living with PD who had cognitive impairment exhibited higher rates of mental health symptoms, including hallucinations and delusions. This co-occurrence of cognitive decline and mental health symptoms presents a unique challenge, as it limits residents’ ability to engage with their environment and caregivers. While quantitative data provides valuable insight into the prevalence and severity of symptoms, qualitative research offers an in-depth understanding of the emotional and social impact. Moreover, Kurtgöz et al., 2024 [26] found that many individuals with Parkinson’s disease (PD) experienced significant spiritual and emotional distress, illustrating the isolation, loneliness, and despair that often accompany the mental health symptoms of PD.

Caregivers frequently experience high levels of emotional and physical strain, which can lead to burnout and deterioration in their own health. One caregiver described the experience as “heart-breaking and exhausting,” [32] highlighting the toll that managing the mental health symptoms of PD can take on family members and caregivers.

Lex et al. [28] highlights a gap in current care models presenting that mental health symptoms are effectively managed but the emotional and psychological distress associated with mental health symptoms continues to undermine the quality of life for many people with PD in care homes. One participant in their study reflected, “Despite all the physical treatments, the constant sadness and anxiety remain a shadow over my day,”. This shows the importance of treating both the motor and non-motor aspects of the disease to improve the overall well-being.

As noted by Porter et al., 2010 [33], people with PD in care homes are typically older, have poorer cognitive function, and are at later stages of the disease, all of which contribute to their increased need for care and supervision. A more integrated and holistic approach to care planning is needed to ensure that both the mental and physical needs of PD residents are met. Across included studies [23,24,25,26,27,28,29,30,31,32,33], mental health and well-being were predominantly assessed via qualitative interviews or observational reports, with comparatively limited use of standardised diagnostic tools or symptom scales.

#### 3.3.2. Theme 2: Experiences of Unmet Mental Health Needs

The studies described substantial unmet mental health needs among residents with Parkinson’s disease living in care homes. Participants reported feelings of isolation, loss of autonomy, and limited emotional support. Accounts frequently depicted environments where mental health symptoms were overlooked or misattributed to the neurological features of Parkinson’s disease. Insufficient staff awareness and time pressures were identified as key barriers to recognising and addressing psychological distress. These findings illustrate the persistent gap between residents’ emotional needs and the support available within care home settings.

One of the primary concerns identified in the literature is the limited expertise among care home staff regarding PD. Van Rumund et al., 2014 [27] conducted a study in Dutch care homes and found that many staff members “lack PD-specific knowledge,” which can lead to the misinterpretation of symptoms and inadequate care. The authors emphasised that the lack of training often results in delays in recognising symptoms such as hallucinations. One key observation from their focus groups highlighted this gap: “nurses were often unaware that the timing of levodopa administration is critical, leading to delays in recognising psychiatric episodes” [27]. This lack of PD-specific knowledge, particularly around the timing of medication, demonstrates the complex interaction between motor and non-motor symptoms that nursing staff often overlook.

Studies by Hosking et al., 2020 [25] and Porter et al., 2010 [33] both reported that care homes are frequently under-resourced, facing high staff turnover and inadequate staffing ratios. Organisational barriers substantially limit the capacity of care teams to provide timely and specialised interventions for managing mental health and well-being in residents with PD. Hosking et al., 2020 [25] found that “inadequate staffing and high turnover mean that even when the need for specialised care is recognised, there is often insufficient time to implement tailored interventions.”

Another crucial barrier to effective management is the lack of integration between different care disciplines. Lex et al., 2018 [28] and Pigott et al., 2023 [31] both reported that there is often insufficient collaboration between general care home staff and specialists, such as neurologists and PD nurses. Lex et al., 2018 [28] found that symptoms were “often untreated due to a lack of streamlined referral processes.” This disconnection between general care staff and specialists often leads to gaps in care, as mental health symptoms remain unaddressed despite their significant impact on residents’ well-being. Furthermore, Pigott et al., 2023 [31] emphasised that residents with PD, who are at higher risk of developing mental health disorders, have “limited access to specialist psychiatric interventions.” The lack of timely and specialised support can lead to the exacerbation of symptoms, which negatively affects residents’ quality of life and functional abilities.

Communication breakdowns further complicate the management of mental health symptoms. Both qualitative and quantitative studies point to recurring issues with communication between staff, residents, and caregivers. Armitage et al., 2009 [29] and Kurtgöz et al., 2024 [26] found that residents and caregivers often expressed frustration with the lack of recognition or acknowledgement of mental health symptoms. For example, one caregiver shared their experience of feeling “unheard, as if our observations of sudden mood swings and hallucinations were dismissed” [29]. This lack of acknowledgment can result in delays in treatment and ultimately in residents suffering from unmanaged symptoms.

The physical environment of care homes also appears to play a role in managing symptoms. Weerkamp et al., 2013 [23] highlighted how environmental factors in care homes, such as the lack of private space and poorly designed communal areas, can exacerbate anxiety and behavioural disturbances in residents with PD. The crowded, noisy, and impersonal environments often found in care homes may increase feelings of stress and confusion for residents, particularly those experiencing hallucinations or cognitive decline. The physical surroundings, therefore, require careful consideration to reduce distress and provide a conducive atmosphere for managing both motor and non-motor symptoms of PD.

A final significant challenge in the management of mental health symptoms, identified in this review, is the continuity of care, especially as PD residents often transition between different levels of care, such as from home-based support to care homes. Research by Pigott et al., 2023 [31] and Porter et al., 2010. [33] indicated that these transitions are often poorly managed, with symptoms under-communicated during the handover process. Pigott et al., 2023 [31] found that “there is a significant risk that NPS are under-communicated, leading to inconsistent treatment plans” during transitions. This discontinuity can result in fragmented care, where residents’ specific needs and histories are not adequately conveyed to the new care team, leading to inconsistent or delayed interventions for symptoms. The lack of a smooth transition between care settings often means that residents may not receive appropriate management for their symptoms, exacerbating the negative impact on their quality of life.

To summarise, this theme has illustrated that the challenges in managing mental health symptoms in PD are deeply rooted in the structural and organisational issues within care home settings [23,24,25,26,27,28,29,30,31,32,33]. Limited expertise among care staff, inadequate staffing, poor integration between general and specialised care providers, communication breakdowns, environmental stressors, and issues with continuity of care all contribute to the difficulty in addressing these complex symptoms. These findings assert the need for a more integrated and well-resourced approach to care, where specialised knowledge, effective communication, and continuity of care are prioritised to better meet the needs of individuals with PD.

#### 3.3.3. Theme 3: Organisational and Systemic Factors Influencing Mental Health Support

The studies identified several organisational and system-level influences on how mental health is recognised and managed in care home settings. Factors such as staff knowledge, training opportunities, communication practices, and inter-professional collaboration shaped the extent to which residents’ mental health needs were addressed. Limited resources, high workload, and the absence of structured screening or referral processes were reported as key constraints. Conversely, supportive leadership and an open organisational culture were described as enabling more responsive and holistic care. Effective mental health support was also linked to wider aspects of Parkinson’s disease management, including timely and consistent medication administration, staff awareness of neuropsychiatric side effects, and access to specialist input when required. These findings emphasise that organisational capacity, training, and clinical governance collectively influence how psychological well-being is supported within care home environments.

Medication management practices were frequently identified as contextual factors that affected residents’ psychological stability. Armitage et al. (2009) [29] described how delayed or inconsistent levodopa administration can exacerbate neuropsychiatric symptoms such as mood changes or hallucinations, particularly in settings with limited staffing or Parkinson’s disease-specific knowledge. Pigott et al. (2023) [31] similarly reported that medication plans tailored to cognitive status reduced behavioural fluctuation, underscoring the importance of staff awareness, training, and timely dosing routines within multidisciplinary care structures.

Alongside pharmacological considerations, psychosocial and multidisciplinary approaches were described as essential components of comprehensive care. Kurtgöz et al. (2024) [26] and Armitage et al. (2009) [29] highlighted the value of structured emotional support activities, peer discussion groups, and recreational engagement in reducing isolation and distress among residents. Van Rumund et al. (2014) [27] emphasised that multidisciplinary teamwork integrating physical, occupational, and psychological support, facilitated by Parkinson’s disease nurse specialists and movement disorder clinicians, promoted coordinated and holistic management.

Emerging technologies were also recognised as potential system-level enablers. Hosking et al. (2020) [25] proposed that telemedicine platforms, remote symptom-tracking applications and wearable sensors could strengthen continuity of care and monitoring where resources are constrained. Pigott et al. (2023) [31] further noted that outcomes vary with cognitive status, reinforcing the need for adaptable care pathways that integrate neurocognitive assessment into ongoing management.

Taken together, these studies demonstrate that the organisation and delivery of care within care homes, including staffing capacity, leadership, interdisciplinary collaboration, training, and access to technology, play a decisive role in determining how mental health support for residents with Parkinson’s disease is achieved [23,24,25,26,27,28,29,30,31,32,33]. The evidence indicates that effective mental health care in this context depends as much on organisational and systemic structures as on individual clinical or psychosocial practices

## 4. Discussion

This review focused exclusively on care home settings and did not address mental health and well-being among individuals with Parkinson’s disease in hospital or community contexts. The findings should therefore be interpreted within this defined scope. While motor symptoms are often the primary focus of PD management, this review reinforces how underappreciated the mental health and well-being of people living with PD is, especially in care homes. Several studies noted that care homes often lack access to specialist input, and staff may misinterpret poorer mental health outcomes as an inevitable part of ageing [34,35]. As Crooks et al., 2025 [36] highlighted, these misattributions can perpetuate stigma and reinforce resident isolation, particularly when behaviours linked to mental health are interpreted as personality traits unrelated to PD. This is supported by findings from studies across multiple international contexts [37,38,39] and clearly reflected in Theme One of this review. Another key finding from this review is related to the systemic and structural challenges within care homes that shape the delivery of care for residents with PD. The findings align with broader literature highlighting the complexity of providing specialist care in these environments, where staff must manage a wide spectrum of conditions with varying trajectories, symptom profiles, and care demands. These difficulties are not unique to PD. Similar barriers have been identified in the management of other long-term and complex conditions within care homes, such as cancer and palliative care. For example, Craig et al.,2023 [40] in their integrative review on cancer care in care homes emphasise how staff frequently lack adequate knowledge, resources, and time to deliver personalised or condition-specific interventions, challenges that are echoed in the PD care context. Likewise, de Campos et al.,2022 [41] argue that delivering high-quality palliative care in care homes is undermined by staff training gaps, competing priorities, and structural under-resourcing. These patterns help contextualise the findings from the current review and highlight how the difficulties in delivering PD care are unsurprising given the broader pressures facing the sector.

In the case of PD, where care must address issues including cognitive decline, mental health disorders, and fluctuating function, such knowledge deficits are particularly detrimental. Theme Two highlighted how these gaps in staff knowledge and confidence often result in reactive rather than proactive care strategies, with a heavy reliance on generalist approaches that may not fully meet the needs of PD residents. This is supported by Finlay et al., 2025 [42] in a recent scoping review of the international literature, which found only seven studies worldwide that evaluated educational interventions on PD in care homes, most of which focused narrowly on motor symptoms. Moreover, the overall quality of the evidence was low, pointing to an urgent need for robust and comprehensive training programmes that involve the full spectrum of Parkinson’s symptoms. Given the range of clinical presentations and the emotional, cognitive, and functional needs of residents with PD, expecting care home staff to have in-depth expertise without substantial support is unrealistic. These professionals are already operating in environments characterised by workforce shortages, high staff turnover, and increasingly complex resident care needs. As such, the findings from this review call for a systems-level response to education, training, and workforce development in care home settings, recognising that staff are doing their best within deeply constrained circumstances [43].

Within the scoping review, some evidence emerged suggesting that targeted therapies, particularly non-pharmacological interventions, can offer promising outcomes for care home residents with PD [26]. These findings show the role of non-pharmacological strategies in addressing both mental health and motor symptoms in PD [23]. However, across the studies reviewed, implementation of such interventions was sparse and often lacked PD-specific tailoring. Several barriers were consistently identified, including the small number of PD residents in each care home, insufficient staff capacity, and practical challenges in maintaining structured programmes such as cognitive stimulation, exercise, or music therapy over time [44]. However, NICE guidelines (2017) [45] strongly recommend the use of non-pharmacological interventions for the management of mood disorders, psychosis, and motor complications in PD, noting their potential to enhance quality of life while reducing reliance on antipsychotics or sedatives. While broader research supports the utility of interventions such as reminiscence therapy, group physical activity, and structured social engagement in improving well-being and mitigating mental health challenges [46,47], the review suggests these are rarely adapted to the specific needs of people with PD in care homes.

Despite the high prevalence of PD and associated complex mental health needs in care home settings, only eleven studies met the inclusion criteria, highlighting a significant gap in the research focused on this population. While several of the included studies were methodologically robust and did include resident voices [26,29,31], the overall volume and scope of literature remain surprisingly limited. Given the established link of disorders such as depression and anxiety in people with PD, and the concentration of frail individuals with complex needs in care homes, a more substantial evidence base would reasonably be expected. This paucity of research reinforces the sense that the care homes remain an overlooked area. Critically, several areas that one would anticipate being addressed in the literature were absent from the findings of this review. For example, there was little exploration of the day-to-day lived experience of PD residents, particularly how mental health symptoms are recognised, interpreted, or responded to by staff, and this is evidenced in a recent review by Copeland et al., 2024 [12], who found that there was also a notable lack of attention to informal carers, family members, or friends, who often remain involved in decision-making even after admission to care homes. This mirrors a recent review from Gonella et al., 2022 [48] about palliative care interventions for family carers in care home settings. In addition, few studies examined the impact of staff training or organisational culture on symptom recognition and management, despite strong evidence from other care contexts that these factors can shape care quality. Multidisciplinary team approaches, widely promoted in PD care pathways for their ability to integrate neurological, psychiatric, and rehabilitative input, were entirely absent from the studies reviewed. Similarly, there was no meaningful focus on how care systems respond to fluctuating symptoms over time, nor any longitudinal data tracking symptom progression, transitions in care needs, or resident outcomes.

There was minimal examination of how race, ethnicity, language barriers, or socioeconomic status may influence symptom reporting, staff response, or resident outcomes, an important oversight, especially in increasingly diverse care home populations [49]. The limited number of studies and narrow focus of existing work highlight how much remains unknown about how best to recognise and respond to mental health symptoms in people with PD in care homes.

Taken together, the findings indicate that mental health support for residents with Parkinson’s disease is fragmented across individual, organisational, and system levels. Psychological needs are often recognised but remain inconsistently addressed because of limited staff capacity, variable training, and absence of integrated care pathways [50,51,52]. Strengthening connections between care home practice and specialist neurological and mental health services may help to bridge this gap. Addressing these systemic gaps requires investment in staff education, clearer guidance, and ethical frameworks that prioritise holistic and person-centred care. Overall, the findings of this review highlight urgent implications across education, research, and practice. As emphasised by Finlay et al. (2025) [42], even basic Parkinson’s disease education can improve symptom identification and staff confidence. International codes of ethics, including those of the International Council of Nurses [53], emphasise that health professionals have a duty to uphold dignity, equity, and informed consent, principles that must guide Parkinson’s disease care in residential settings. This scoping review also reveals a near-total absence of research examining how mental health and well-being are interpreted and managed by care home staff, underscoring the need for targeted investigation in this area.

To our knowledge, no scoping or systematic reviews have specifically examined the mental health and well-being of people with Parkinson’s disease residing in care homes. Existing reviews have focused on community-dwelling individuals or on non-motor symptoms more broadly [12,42]. The present review therefore complements and extends previous syntheses by focusing specifically on institutional care settings.

### Strengths and Limitations

The inclusion criteria were designed to capture studies that explicitly focused on care home residents with a PD diagnosis and reported on mental health and well-being. Although this deliberate focus found only 11 studies, it ensured strong alignment with the review’s aim. However, it may have limited the overall number of eligible studies.

As scoping reviews aim to map the breadth of existing evidence, studies older than 15 years [24,29] were included to provide valuable context in a field where research remains limited.

Several limitations should be noted. First, limiting the search to English-language studies may have introduced language bias. This restriction was necessary due to limited translation resources, which may have resulted in the review primarily reflecting a Western European perspective. Second, heterogeneity in outcome measures across the included studies complicated direct comparisons. Finally, some studies relied on caregiver-reported data, which may have introduced bias, particularly in cases where residents with advanced PD were unable to self-report [24,30].

## 5. Conclusions

Poor mental health can impair quality of life for care home residents with PD, exacerbating emotional distress and social isolation. To advance care quality within care homes, staff need to be proactive in the development of initiatives in promoting mental health and well-being in residents. Education and training programmes should be embedded within care home staff’s routine continued professional development and modules with specific focus on Parkinson’s. We, the authors, advocate for care homes to incorporate routine mental health screening and to incorporate regular assessment of mood, anxiety, and psychotic symptoms alongside physical health checks and use validated screening tools where possible (e.g., NPI for neuropsychiatric symptoms).

Future research should focus on actively engaging individuals with PD to understand what they would want from these care homes to optimise their mental health and well-being. Addressing these gaps requires coordinated organisational strategies, enhanced staff competencies, and integrated models of care to support the mental health and well-being of residents with Parkinson’s disease.

## Figures and Tables

**Table 1:** PEO framework and search terms.

Population	(MH “Parkinson Disease”) OR “Parkinson’s Disease” OR PD OR “Parkinsonian” OR “Primary Parkinsonism” OR “Paralysis Agitans” OR “Progressive Supranuclear Palsy” OR “Multiple System Atrophy of Corticobasal Degeneration”
Exposure	(MH “Nursing Homes”) OR “Care home” OR “Care homes” OR “Nursing home” OR “Residential home” OR “Residential homes” OR “Long term care facility” OR “Residential Aged Care Facility” OR “Long term care facilities” OR “Homes for the Aged”
Outcome	(MH “Depression”) OR (MH “Anxiety”) OR (MH “Quality of Life”) OR (MH “Hallucinations”) OR “Low mood” OR (MH “Symptom Burden”) OR (MH “Psychological Well-Being”) OR “emotional well-being” OR “experience”

**Table 2:** Inclusion and exclusion criteria.

Criterion	Inclusion	Exclusion
Population	Individuals diagnosed with PD residing in care homes (e.g., residential, nursing, or long-term care facilities)	Individuals with other neurodegenerative conditions (e.g., dementia) unless data for PD are separately reported
	Caregivers (formal or informal) of individuals with PD with care home settings (studies including caregivers or professionals were eligible only when providing data relevant to the mental health or well-being of residents with Parkinson’s disease)	Studies that do not involve individuals with PD or their caregivers
	Health or social care professionals involved in the care of individuals with PD in care homes	
Exposure	Mental health (e.g., depression, anxiety, psychosis, hallucinations, apathy, agitation, sleep disturbances, cognitive impairment)	Studies focusing exclusively on motor symptoms or unrelated health issues
Setting	Care home settings, including residential care, nursing homes, and long-term care facilities	Community-based or outpatient settings, hospitals, or acute care facilities
Outcomes	To better understand the state of mental health and well-being among residents with Parkinson’s disease and care delivery	Studies not reporting outcomes related to mental health and well-being
Study Design	Qualitative, quantitative, and mixed-methods studies	Editorials, commentaries, opinion pieces without primary data
Language	English-language publications	Non-English publications without available translation
Publication Date	No restrictions	

**Table 3:** Summary of review findings.

Study	Aim	No. of Participants	Setting	Method of Data Collection	Key Findings
Aarsland et al. (1999) [24]	To assess the range of mental health symptoms in a representative sample of patients with Parkinson’s disease.	139 A structured interview was conducted with the caregiver of each patient (not the patient themselves) to complete the NPI. ~36 staff members (one per nursing home resident) ~103 family caregivers (for home-dwelling patients).	Nursing homes	Structured interviews with caregivers and patients	A strong association was found between the severity of mental health (such as depression, anxiety, and hallucinations) and the advanced stage of Parkinson’s disease, indicating that these symptoms significantly contribute to reduced well-being in PD patients.
Armitage et al. (2009) [29]	To explore and describe the care of persons with Parkinson’s disease (pwPD) who are care home residents.	24 residents living with Parkinson’s disease across 43 care homes. 45 care plans were analysed to assess how care was documented and planned for pwPD residents.	Care homes	Interviews, review of care plans	Only 13 out of 45 care plans (28%) were accessible to residents or relatives. Only 6 care plans were co-signed by pwPD or relatives. The level of specific written detail about PD was minimal when compared to the relative’s descriptions of the affected person’s needs.
Chang et al. (2023) [30]	To investigate the prevalence of risk factors for mental health symptoms in institutionalised patients with PD in Taiwan.	370 Patients with Parkinson’s disease (pwPD): A total of 370 institutionalised patients with a confirmed diagnosis of Parkinson’s disease were included in the final analysis. These participants were drawn from 266 long-term care service institutions across 22 cities and counties in Taiwan. Caregivers (staff): Although the exact number of caregivers is not stated, each of the 370 patients had at least one caregiver informant involved.	Institutions	Survey conducted in two stages	Depression (14.3%) and anxiety (13.2%) were among the most common disorders in institutionalised PD patients. The study emphasised the need for regular screening and targeted interventions for this population.
Hosking et al. (2020) [25]	To determine clinical characteristics and treatment complications of patients with late-stage parkinsonism living in nursing homes compared to those living at home.	692 Total participants: 692 patients with late-stage parkinsonism Nursing home residents: 194 Living at home: 498 Caregivers: All participants had a caregiver involved (either a family member or professional care staff), as data collection included patient and caregiver input.	Nursing homes	Structured interviews and validated assessment tools	Only cognitive function (MMSE, *p* = 0.03) and mental health symptoms (NPI-Q, *p* = 0.01) were significantly worse among nursing home residents, while other measures such as motor severity (MDS-UPDRS-III), depression, anxiety, and dementia staging showed no statistically significant differences (*p* > 0.05).
Kurtgöz and Genç (2024) [26]	To determine the perceptions and experiences of elderly PD patients and caregivers regarding spiritual care.	23 All 23 participants were interviewed, 8 residents Originally, 14 were approached 3 were excluded due to advanced dementia 3 were excluded due to communication difficulties 15 caregivers were interviewed	Nursing homes	Face-to-face semi-structured interviews interviewed Interviews took place in counselling rooms within the nursing homes	Profound sense of psychological distress among both residents and caregivers, though the exact number of participants reporting this theme was not specified. One resident remarked, “I yearn for spiritual support that makes me feel whole, yet it is never offered here.”
Lex et al. (2018) [28]	To examine the medical and nursing demands of residents in late-stage Parkinson’s disease cared for in residential homes.	9 residents with PD who required support in activities of daily living and five family members.	Nursing homes	Semi-structured ethnographic interviews	All nine participants exhibited severe limitations in daily activities, but some residents reported a degree of contentment with their living situations. This variability in personal coping strategies was evident despite the physical challenges of late-stage PD.
Pigott et al. (2023) [31]	To compare care needs and resource use for PD patients with and without cognitive impairment.	675 Total participants living in a care home or similar setting: 177 out of 675 participants (26.2%) The patient, where feasible A caregiver— either: An informal caregiver (e.g., spouse, child) if the patient lived at home A professional caregiver (e.g., care home staff) if the patient lived in a care home	Community settings	Structured face-to-face interviews with both patients and their caregivers	The study found that patients with cognitive impairment were less likely to consult PD nurses, indicating that cognitive deficits may serve as a barrier to accessing specialised PD care, highlighting the need for tailored care strategies for this group.
Porter et al. (2010) [33]	To assess the care requirements of people with idiopathic Parkinson’s disease (PD) and compare them with a similarly aged background population.	135 patients (83.8%) agreed to participate in the study. 19 patients (14.1%) were living in residential or nursing homes. 116 patients (85.9%) were community-dwelling.	Residential care	Structured questionnaire The study relied entirely on patient self-report, and some data were excluded when cognitive impairment made self-report unreliable.	Patients with cognitive impairment were older, had worse motor scores, and were more likely to live in care homes. Those experiencing visual hallucinations had more advanced disease and poorer quality of life but were equally as likely to live at home as those without hallucinations.
Van Rumund et al. (2014) [27]	To explore the unmet needs of nursing home residents with Parkinson’s disease (PD) and assess care quality from the perspectives of residents, caregivers, and healthcare workers.	Patients with Parkinson’s disease (PD) or parkinsonism interviewed: 15 nursing home residents 15 informal caregivers (not related to the 15 patients, to ensure diverse perspectives) Focus group discussions were used for: The 35 health care professionals	Nursing homes	Semi-structured interviews were used for: All 15 residents All 15 informal caregivers Five focus groups were conducted: Two with nurses Three with multidisciplinary professionals Each focus group lasted approximately 2 h and followed a topic guide.	Participants (residents, caregivers, and healthcare staff) reported several unmet needs, such as inadequate emotional support and insufficient PD-specific knowledge among staff. These unmet needs highlighted gaps in patient-centred care and the importance of addressing mental health symptoms of PD.
Walker et al. (2013) [32]	To investigate factors leading to institutionalisation of PD patients and ongoing care needs in care homes.	90 patients with idiopathic Parkinson’s disease or other forms of parkinsonism were included. Types of care: 31 residents (34.4%) were in residential care 48 (53.3%) in nursing care 11 (12.2%) in mixed residential/nursing care	Care homes	Retrospective observational study, using: Review of medical records Documentation of: Hospital attendances Admissions Medication use Disease characteristics Reasons for admission to care Time frame: January 2011 to December 2012 (2-year period before the audit in 2013)	Over one-third of patients were taking antidepressants. Depression in PD was found to be undertreated, with antidepressants potentially increasing fall risks in elderly individuals, already a concern in PD.
Weerkamp et al. (2013) [23]	To determine the prevalence of non-motor symptoms in nursing home residents with Parkinson’s disease (PD) and to establish the association with quality of life.	While 73 residents were personally assessed through structured interviews and tests, family or staff were not formally interviewed. Nurse-reported tools like the NPI-NH were used for some symptom assessments.	Nursing homes	Cross-sectional study, standardised assessments	Individuals with PD experienced an average of nearly 13 non-motor symptoms per person, with depressive symptoms observed in 45%. The total non-motor symptoms burden was significantly associated with reduced quality of life (R^2^ = 0.39).

## Data Availability

No new data were created or analysed in this study.

## Abbreviations

The following abbreviations are used in this manuscript:

PD	Parkinson’s Disease
pwPD	People with Parkinson’s Disease
NPS	Neuropsychiatric symptoms
MMAT	Mixed Methods Appraisal Tool
